# Syndecan-1 in Liver Diseases

**DOI:** 10.1007/s12253-019-00617-0

**Published:** 2019-03-02

**Authors:** Eszter Regős, Katalin Karászi, Andrea Reszegi, András Kiss, Zsuzsa Schaff, Kornélia Baghy, Ilona Kovalszky

**Affiliations:** 1 1st Department of Pathology and Experimental Cancer Research, Semmelweis University, Üllői Street 26, Budapest, 1085 Hungary; 2 2nd Department of Pathology, Semmelweis University, Üllői street 93, Budapest, 1091 Hungary

**Keywords:** Syndecan-1, Liver, Cirrhosis, Metastasis, Hepatocellular carcinoma

## Abstract

Liver diseases such as liver cirrhosis, primary and metastatic liver cancers are still a major medical challenge. Syndecan-1 is one of the most important proteoglycans in the liver. Syndecan-1 is normally expressed on the surfaces of hepatocytes and cholangiocytes. Due to liver diseases the amount of syndecan-1 increases in the liver. Despite the emerging data of the biological function of syndecan-1, the clinical usefulness of this proteoglycan is still unknown. In our study we correlated syndecan-1 expression to clinico-pathological data. We found that syndecan-1 proved to be a good marker for hepatitis C virus based hepatocellular carcinoma and increased with liver dysfunction.

## Introduction

Despite of the permanent progress in the understanding of mechanisms implicated in the development of liver diseases, the management of liver cirrhosis and malignant liver tumors are still a major medical challenge. The most important etiology factors of the primary liver diseases, including liver fibrosis and hepatocellular carcinomas (HCC), are hepatitis B and C viral infections (HBC, HCV) and alcohol consumption [1]. However, the liver is more frequently target of metastatic than primary tumors. These are mainly adenocarcinomas arisen from various tumors of the body, but the most common place of origin is colorectal cancer [2]. Tumors, developed in non-cirrhotic background can be treated by partial resection of the liver. As cirrhosis destroys the liver parenchyma, liver transplantation is the only effective therapeutic approach either without or with the presence of HCC. Here, in order to achieve the most effective organ allocation, objective scoring systems were necessary to develop. Recently, the most widely used system is the MELD (Model For End-Stage Liver Disease) score. MELD score is based on three entirely objective laboratory test, namely serum total bilirubin level, serum creatinine level and International Normalized Ratio (INR) [3].

In the meantime, liver injury is accompanied by deterioration of several other laboratory parameters. Among those we observed the changes of syndecan-1 (SDC1). This molecule, a member of the syndecan family, is the major proteoglycan of the liver, built up by and extracellular, a transmembrane and an intracellular domain. In proteoglycans glycosaminoglycans are covalently attached to the core protein. In case of syndecan-1 three heparan-sulphate and two chondroitin-sulphate side chains can be detected on the core protein. The extracellular domain of syndecan-1 interacts with numerous types of ligands, including growth factors, cytokines, receptors and enzymes [4]. Physiologically syndecan-1 is predominantly expressed on the surface of epithelial cells, but is also present on pre-B and plasma cells [5]. It has well-documented roles in wound repair, development, stem cell differentiation, inflammation and tumorigenesis [4, 6, 7]. Another characteristic of syndecan-1 is the shedding of the extracellular domain. SDC1 can be cleaved at the juxtamembrane site by various proteolytic enzymes, putting the extracellular domain in soluble form [6, 8]. The serum concentration of the shed extracellular domain increases in liver diseases such as non-alcoholic fatty liver disease and liver fibrosis, reported to be useful biomarker for disease monitoring [9, 10].

According to our previous studies the amount of heparan-sulphate and chondroitin-sulphate increases in various primary liver diseases, associated with altered expression of syndecan-1 [11, 12]. However, there is no indication in human diseases whether changes in syndecan-1 expression indicate protective or deleterious action?

Our aim was to correlate the amount of syndecan-1 in primary liver diseases and colorectal adenocarcinoma metastasis with relevant clinical data.

## Materials and Methods

### Patient Samples

The liver specimens for our study were taken from the archives of the 2nd Department of Pathology and from the 1st Department of Pathology and Experimental Cancer Research Semmelweis University. All experiments were conducted according to the ethical standards of Hungarian Medical Research Council, Budapest, Hungary (permit no. TUKEB 95/1999, 2/2012).

Non-tumorous part of surgically removed liver hemangiomas served as control normal liver samples. In case of hepatocellular carcinomas the patients underwent only partial liver resection. The tumor and its non-tumorous adjacent areas (NTA) were parallel analyzed. The samples from the liver cirrhosis were obtained after liver transplantations. In this case two representative cores were examined.

In case of colon adenocarcinoma primary tumors and their surrounding non tumorous colon tissues were compared to the paired metastasis and its non-tumorous peritumoral livers.

The sample distribution and the number of the cases are described in Table 1.

**Table 1:** Number of cases involved in the study

Control liver sample	9
HCC non-cirrhotic based	9
HCC cirrhotic based	19
Cirrhotic liver samples	29
Alcoholic liver disease: 13	
Hepatitis B virus: 4	
Hepatitis C virus: 12	
Colon carcinoma and metastasis metachron tumor	43
Colon carcinoma and metastasis synchron tumor	38

### Syndecan-1 Immunhistochemistry

Formalin-fixed paraffin-embedded sections were dewaxed in xylene and ethanol, and subsequently washed with dH_2_O. Antigen retrieval was performed in a pressure cooker using TRIS-EDTA buffer (10 mM TRIS, 1 mM EDTA, 0,05% Tween20, pH = 9) for 30 min. After cooling slides were washed with PBS, and endogenous peroxidase was inactivated by addition of 10% H_2_O_2_ in methanol for 20 min. To prevent any nonspecific binding 5 *w*/*v*% bovine serum albumin (BSA) containing 10 *v*/v% normal serum dissolved in PBS was applied for 1 h at room temperature. Monoclonal Mouse anti-human CD138 Clone M15, (Dako Agilent, Santa Clara, CA) diluted at 1:50 in 1 *w*/*v*% BSA/PBS was applied overnight at 4 °C. The next day, slides were washed in PBS containing 0,05% Tween-20, then horse radish peroxidase conjugated Polyclonal Goat anti-Mouse Ig HRP (Dako Agilent) at 1:200 dilution was used for 1 h at room temperature. The immunoreaction was detected by Novolink DAB Substrate Buffer, according to the manufacturers recommendation (RE 7143, RE 7105, Leica Biosystem, Wetzlar Germany) [13].

### Digital Analysis

The slides were scanned using Pannoramic 250 digital scanner (3DHistech Ltd., Budapest, Hungary). The samples were analyzed by the QuantCenter image analysis software’s DensitoQuant modul. Briefly, the module measures the density of immunoreaction on the digital images by distributing pixels to negative and 3 grades of positive classes by their RGB values. In case of cirrhotic and tumorous liver samples, the ratio of strong and moderate pixels was counted and was compared to the whole examined area. For colorectal carcinoma and liver metastasis samples, H-score („histo”-score) was calculated using the following formula: [1*(% weak positive areas) + 2*(% moderate positive areas) + 3*(% strong positive areas)]. Both measurements were applied for the normal, control liver tissue as well. This distinction in the analytic method was necessary since the large number of syndecan-1 positive plasma cells, physiologically present in the lamina propria of the colon mucosa, had to be excluded from the analysis.

### Statistical Analysis

All statistical analyses were made with GraphPad Prism 6.01 software (Graphpad Software Inc.). Significance levels were tested by non-parametric tests (Mann-Whitney) or Students’ t-tests depending on the distribution of the data. Significance levels were selected as *:*p* < 0,05, **:*p* < 0,01 and ***:*p* < 0,001.

## Results

### Syndecan-1 in Normal Liver

**Fig. 1:**
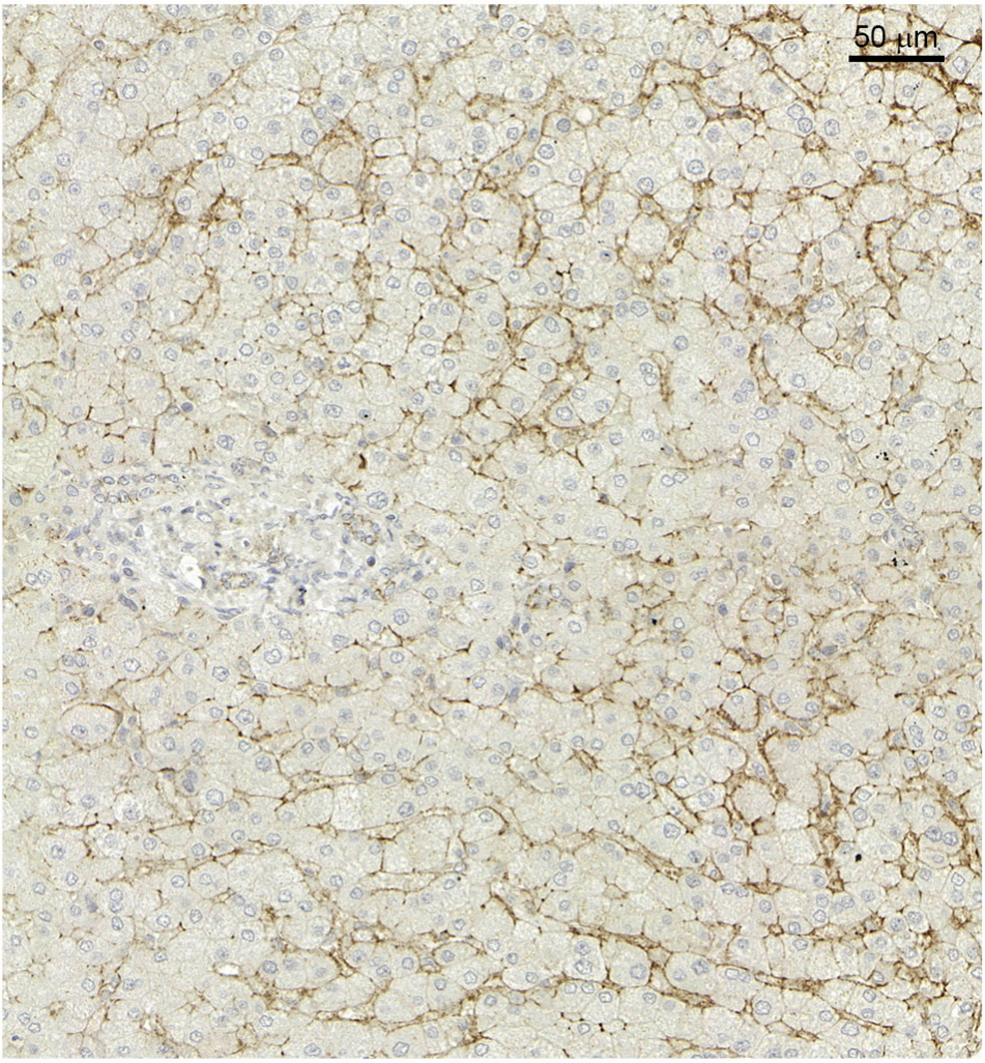
Syndecan-1 immunhistochemistry in normal liver. Scale bar: 50 μm

In the control liver samples syndecan-1 is expressed on the basolateral surface of the hepatocytes. By this localization, it marks the sinusoids of the liver. The intensity of the reaction is modest (Fig. 1).

### Syndecan-1 in Liver Cirrhosis

**Fig. 2:**
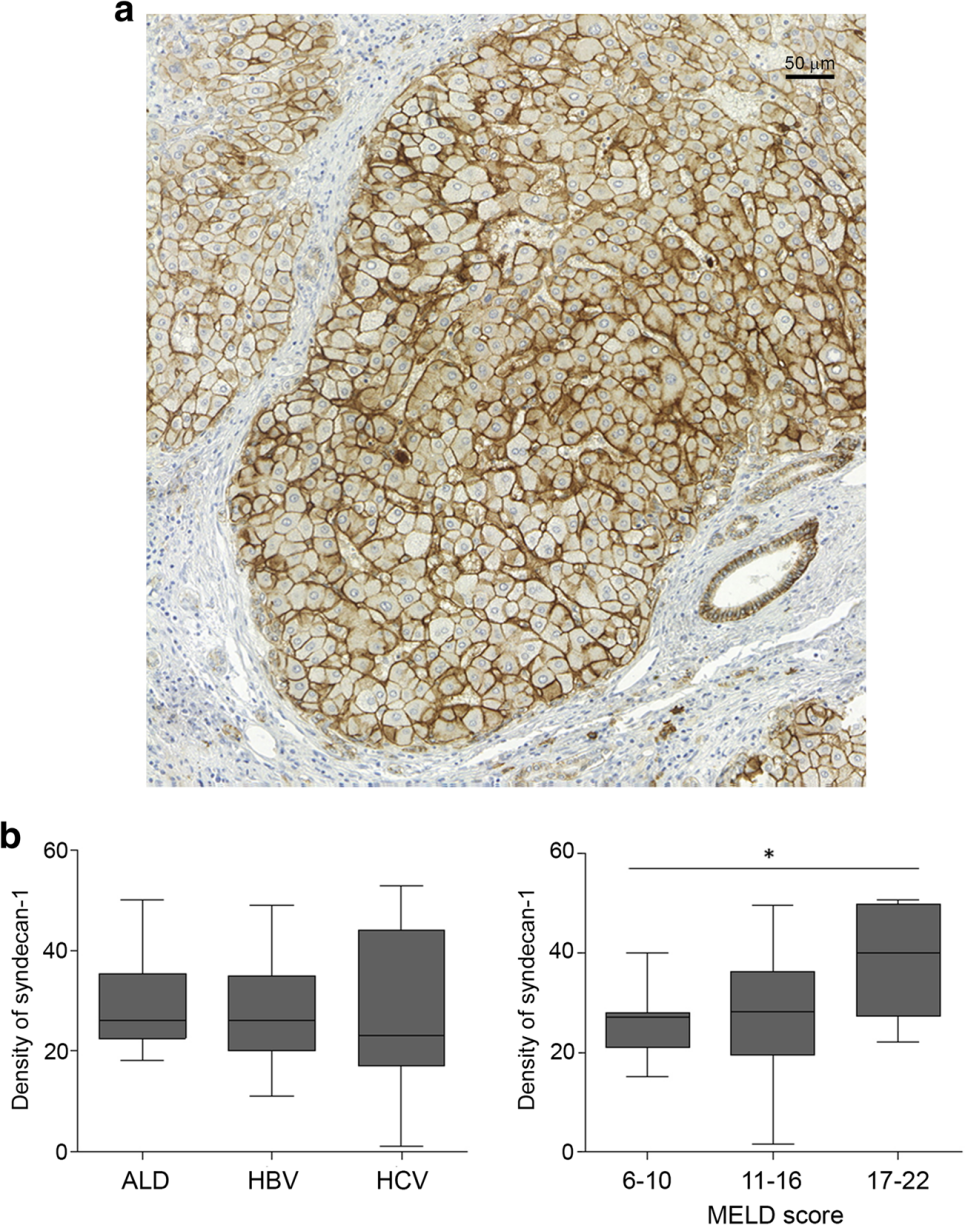
**a** Syndecan-1 immunhistochemistry in cirrhotic liver samples. Scale bar: 50 μm **b** Results of image analysis. ALD: Alcoholic liver disease HBV: Hepatitis B virus infection HCV: Hepatitis C virus infection

Compared to normal control liver samples, the amount of syndecan-1 is significantly enhanced in cirrhotic liver samples. The polarized distribution of the proteoglycan disappeared, strong circumferential reaction on the plasma membranes, surrounded each individual hepatocytes, was observed. Strong immunopositivity was present between the lateral surfaces of the cells of the proliferating bile ducts (Fig. 2a).

Disappointingly the amount of syndecan-1 did not show any connection with etiology of liver cirrhosis (Fig. 2b). On the other hand, it related to the severity of the disease, significantly increasing in parallel with the MELD score.

### Syndecan-1 in Hepatocellular Carcinoma and Non-Tumorous Adjacent Area

**Fig. 3:**
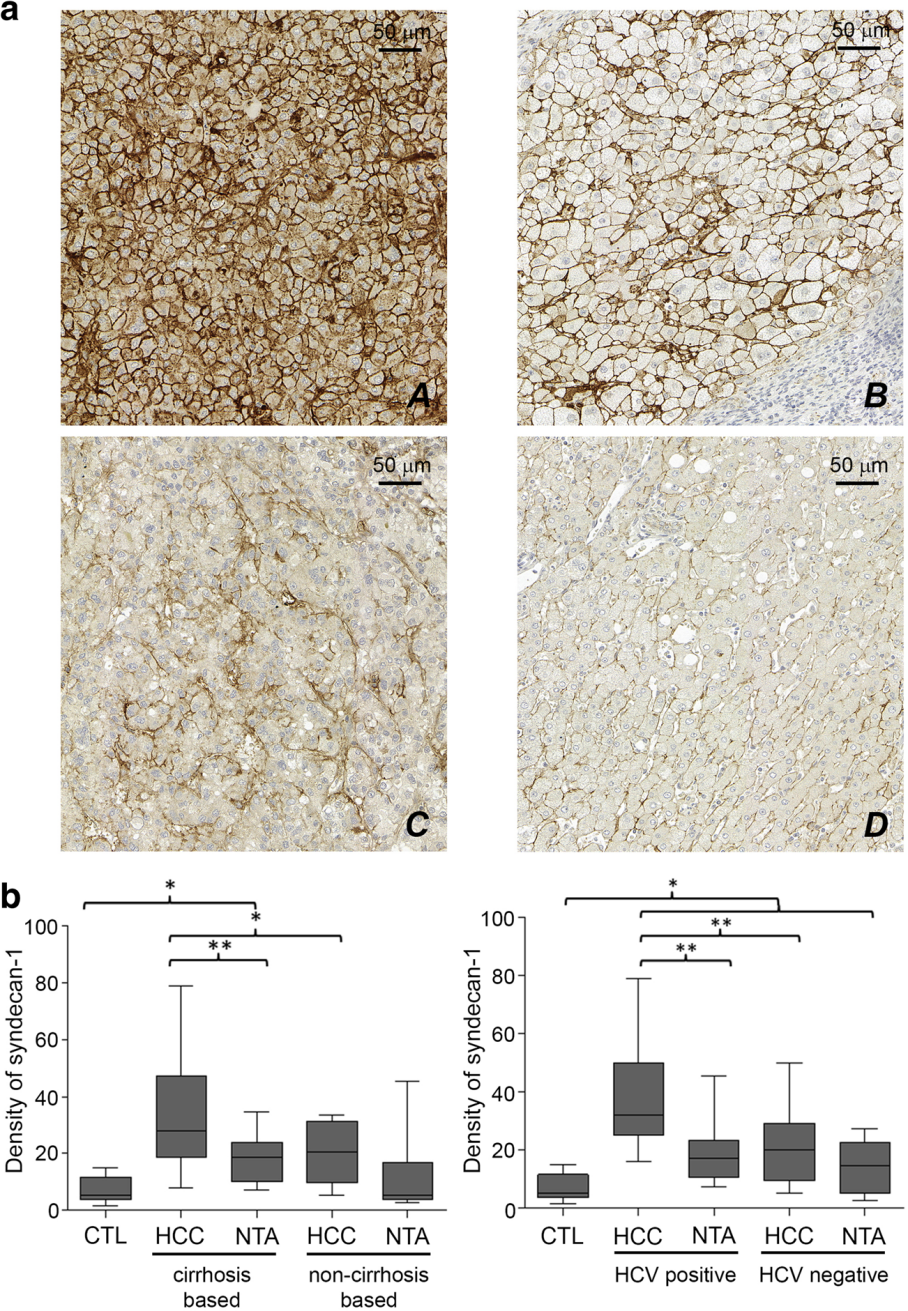
**a** Representative images of each group. A: Hepatocellular carinoma based on cirrhosis. B: Non-tumorous adjecent areas based on cirrhosis C: Hepatocellular carcinoma without underlying cirrhosis. D: Non-tumorous adjecent areas without underlying cirrhosis. **b** Results of image analysis. HCC: heparocellular carcinoma. NTA: non-tumorous adjecent areas HCV: hepatitis C virus

The amount of syndecan-1 in liver carcinoma samples depended on the previous pathology of the organ. Modest increase could be found when the tumor developed in non-cirrhotic liver, whereas strong circumferential positivity of the plasma membrane characterized the tumor cells, developed in cirrhotic livers. Immunopositivity of the adjacent peritumoral cells lagged behind those of tumor tissue. Fibroblast and the scar tissue do not show syndecan-1 expression (Fig. 3a).

We analyzed the correlation between the level of expression and various clinical parameters, such as grade or stage of tumor. With more advanced tumor stage the level of syndecan-1 proved to be higher, however, the difference did not proved to be significant (data not shown). On the other hand, if we took the etiology into consideration, hepatitis C positivity went together with significant elevation of syndecan-1 (Fig. 3b).

### Syndecan-1 in Primary and Metastatic Colon Adenocarcinoma

**Fig. 4:**
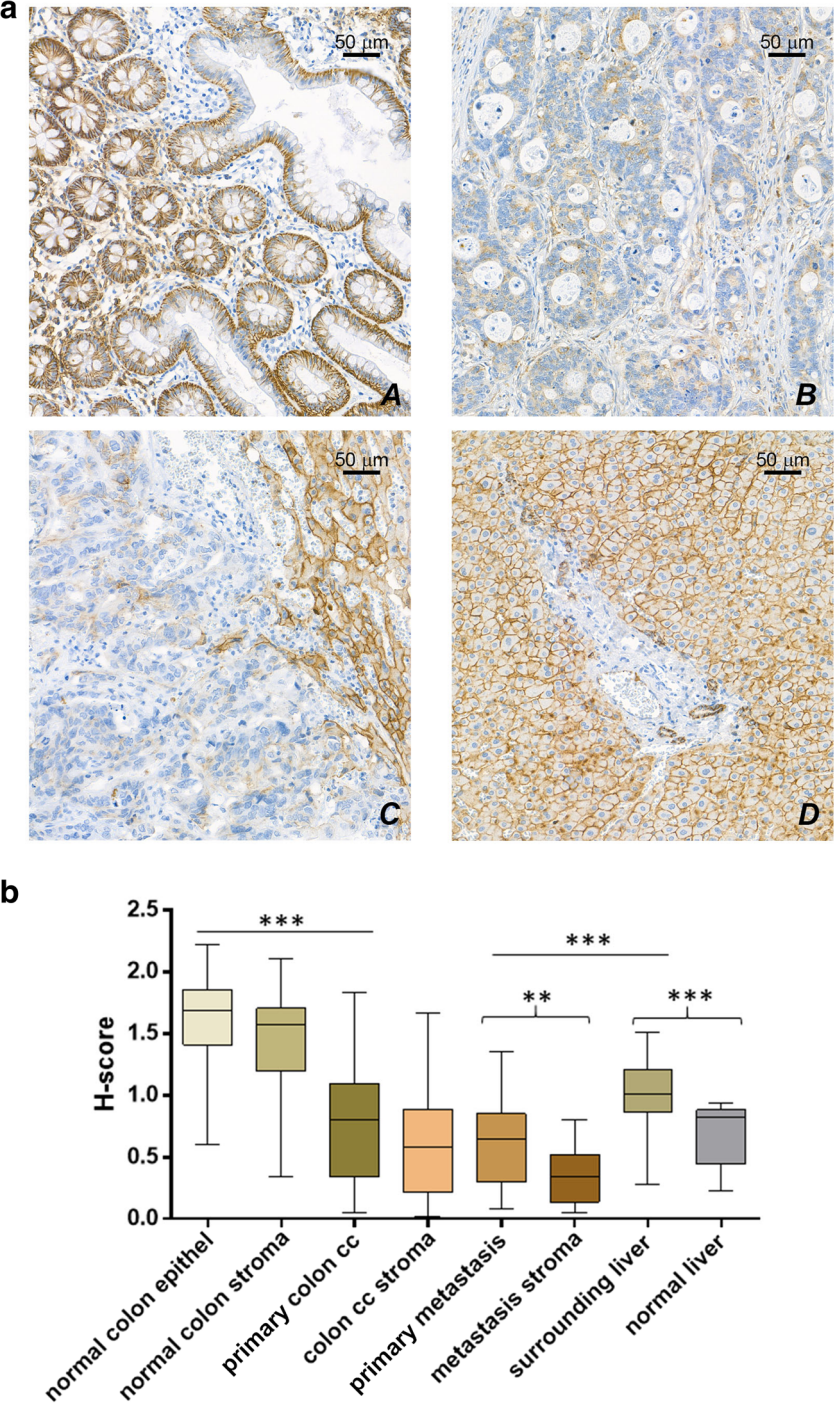
**a** Representative images of each group. A: Normal colon tissue B: Colon adenocarcinoma C: Colon adenocarcinoma liver metastasis D: Non metastatic tumorous adjecent areas of the liver **b** Results of image analysis

On the normal colon epithelium syndecan-1 immunreaction showed strong circumferential positivity, in addition small cells with syndecan-1 positive plasma membrane could be detected in the lamina propria, indicating there the presence of plasma cells. Compared to normal colon epithelium the amount of syndecan-1 proved to be significantly reduced in primary colon adenocarcinomas (Fig. 4a and b). The primary and metastatic colon adenocarcinoma cells and also their neostroma retained their low syndecan-1 expression. This phenomenon was observed both in synchron and metachron cases. With image analysis we did not find significant difference between the primary tumor and its liver metastases what syndecan-1 expression concerns. Interestingly the neostroma of metastasis (mostly containing fibroblasts) are almost negative considering syndecan-1 expression. The hepatocytes adjacent to the metastatic nodules showed significantly increased syndecan-1 immunoreactivity compared to the control liver cells (Fig. 4a and b). We did not find any correlation between T and N stage of the primary colon adenocarcinoma and the amount of syndecan-1 expression. (Data not shown.)

## Discussion

Syndecan is the major heparan-sulphate proteoglycan of the liver. Although its function in various physiological and pathological events is excessively studied we are far for understanding its role in liver diseases. This proteoglycan plays critical role in the clearance of lipoproteins from the circulation, acting as a VLDL receptor [14]. A typical feature of syndecan pathologies is the shedding of its extracellular domain, which subsequently appears in the circulation. Really several publications described elevated serum syndecan-1 level accompanying the deterioration of the liver [9]. This also implies, that assessment of liver syndecan as marker of the liver disease should calculate with the cell surface and shed syndecan-1 together.

Previously [11] and now in this present systematic study we demonstrated that the pathological events of the liver tissue is always related to the alteration of syndecan-1 expression. Recently we described, that increased expression of the proteoglycan triggers the synthesis of MMP-14, a protease playing critical role in matrix degradation, hindering this way the development of liver cirrhosis in experimental system [13].

Syndecan-1 as a transmembrane cell surface molecule is capable to initiate crosstalk between the extracellular and intracellular milieu, thus it is an important player of tissue communication, and co-receptor of cell surface TK receptors [15, 16]. Furthermore, it is involved in calcium signaling [17]. Now the question is, how can we translate the experimental data to the human liver diseases?

In this manuscript we focused on the changes of syndecan-1 developed in hepatocellular carcinoma with and without cirrhosis, as well as on its change as the response of tumor metastasis.

As we demonstrated the amount of syndecan-1 varies in primary and metastatic liver diseases compared to normal controls. In the different primary liver diseases like liver fibrosis or hepatocellular carcinoma we have found elevated syndecan-1 levels. The most prominent enhancement of syndecan-1 expression could be observed in cirrhosis based hepatocellular carcinoma and cirrhosis. Sorting out the factors responsible for this upregulation HCV turned out to be responsible for it. Really, it was published, that syndecan-1 is the receptor of HCV and other viruses as well [18]. In the meantime this over production of syndecan-1 goes together with its increased shedding, which seemed to be beneficiary in experimental liver fibrosis.

Our experimental data indicated, that syndecan-1 overexpression inhibits the early stages of liver fibrogenesis through its shedding. The shedded extracellular domain of syndecan-1 binds and eliminates TGFβ1, one of the most important cytokines in fibrogenesis, to the circulatory system [13]. In this study both syndecan-1 and TGFβ1 concentration proved to be elevated in the sera in accordance with previous publications demonstrating that syndecan-1 increased in the patients’ sera parallel with the severity of the disease [9, 10].

For liver diseases like liver fibrosis still only transplantation is the curative solutions. In order to establish the best organ allocation objective decision making is necessary. In the decision making process MELD score is a helpful tool to establish the severity of the disease and predicts 3-month mortality among patients with chronic liver disease waiting for transplantation [19]. In in vivo experiment syndecan-1 attenuated acetaminophen induced acute liver failure and promoted liver repair in mice [20]. In our study enhanced MELD score groups went together with higher amount of syndecan-1 on hepatocyte surface. These data suggests that although syndecan-1 has a protective role in liver fibrosis and liver failure, this defense mechanism can exhaust after a while.

Compared to normal liver areas increased amount of syndecan-1 could be observed both in cirrhosis and non-cirrhosis based hepatocellular carcinoma, however it is much higher in the former, where syndecan-1 expression is also high in the peritumoral cirrhotic areas. Our results showing increased syndecan-1 expression in HCV infected specimens were full agreement with the previous studies describing that syndecan-1 and its heparan-sulphate glycosaminoglycan chain is a major receptor for HCV internalization to hepatocytes [18, 21].

Previous studies have already proved that syndecan-1 expression is down regulated in case of colon adenocarcinomas compared to normal colon epithelium, and this has a strong association with the size of the tumorous tissue [22]. In our studies we have also observed the same decrease in case of colon samples, and similarly low amount of it was observed in their liver metastases either synchron or metachron cases. If we compared the non tumorous surrounding liver samples to normal liver higher amount of syndecan-1 can be observed. It is a question to address if in this scenario the elevated syndecan-1 level is part of the defensive mechanism or the tumorous niche?

In **conclusion** we have found that the amount of syndecan-1 increases in the different liver diseases compared to normal samples, which, based on the experimental data may indicate defense mechanism, however permanent injuries result in the final exhaustion of these protective processes with final exhaustion of the liver function.

## Abbreviation

HBV: Hepatitis B virus
HCV: Hepatitis C virus
HCC: Hepatocellular carcinoma
INR: International Normalized Ratio
MELD: Model For End-Stage Liver Disease
NTA: Non-tumorous adjacent areas
SDC: Syndecan-1
